# Calcifying fibrous tumor of the small intestine

**DOI:** 10.1093/jscr/rjaf158

**Published:** 2025-03-26

**Authors:** Zouhry Ibrahim, Achraf Bahi, Ghannou Nizar, Halim El Mustapha

**Affiliations:** National Institute of Oncology, Av.Allal Al Fassi Rabat, B.P. 10100, Morocco; Department of Visceral Surgery, HMIMV, Av.Abderrahim Bouabid Rabat, B.P. 10040, Morocco; National Institute of Oncology, Av.Allal Al Fassi Rabat, B.P. 10100, Morocco; Department of Visceral Surgery, HMIMV, Av.Abderrahim Bouabid Rabat, B.P. 10040, Morocco

**Keywords:** calcifying fibrous tumor, small intestine, surgery

## Abstract

We report an observation of a calcifying fibrous tumor discovered through imaging in a 28-year-old male presenting with chronic abdominal pain associated with diarrhea. The abdominal computed tomography scan revealed an intraperitoneal mass located in the right iliac fossa, containing calcifications and measuring 37 × 49 mm; biopsies were non-contributory. A diagnosis of a suspected gastrointestinal stromal tumor was made, and an ileocecal resection was performed. Histological examination confirmed the diagnosis of a calcifying fibrous tumor. The patient was doing well without recurrence six months post-surgery. Only a few observations of calcifying fibrous tumors have been reported in the literature. These benign tumors are often discovered incidentally and have fairly characteristic clinical and pathological features that allow them to be distinguished from stromal tumors.

## Introduction

The calcifying fibrous tumor is a rare benign tumor with various locations in the body. The ileal localization is rare, with only a few cases reported in the literature. We present a case of a calcifying fibrous tumor of the ileum in a young individual and discuss the diagnostic and therapeutic challenges of this rare pathology.

## Case presentation

**Figure 1:**
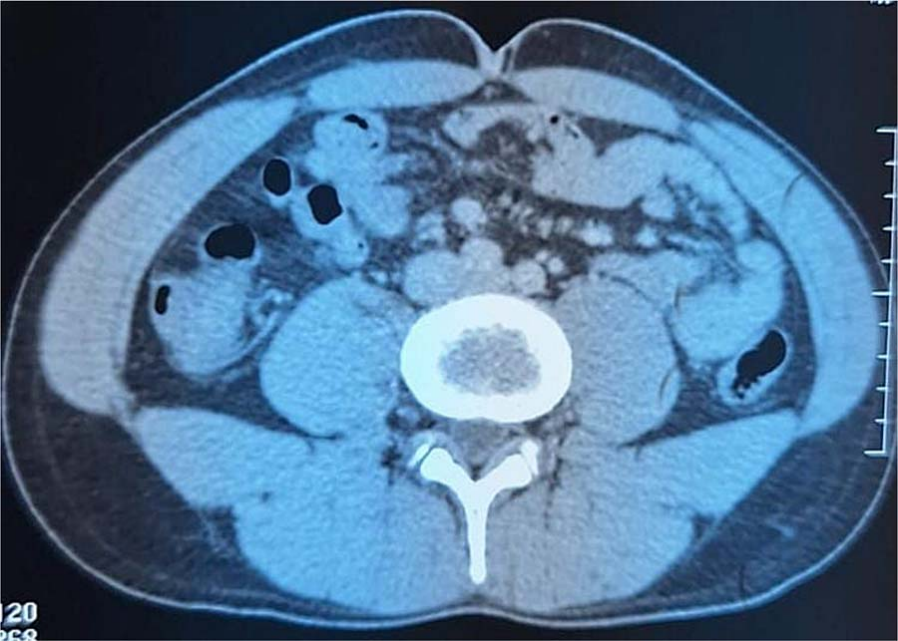
Intraperitoneal mass located in the right iliac fossa, containing calcifications.

**Figure 2:**
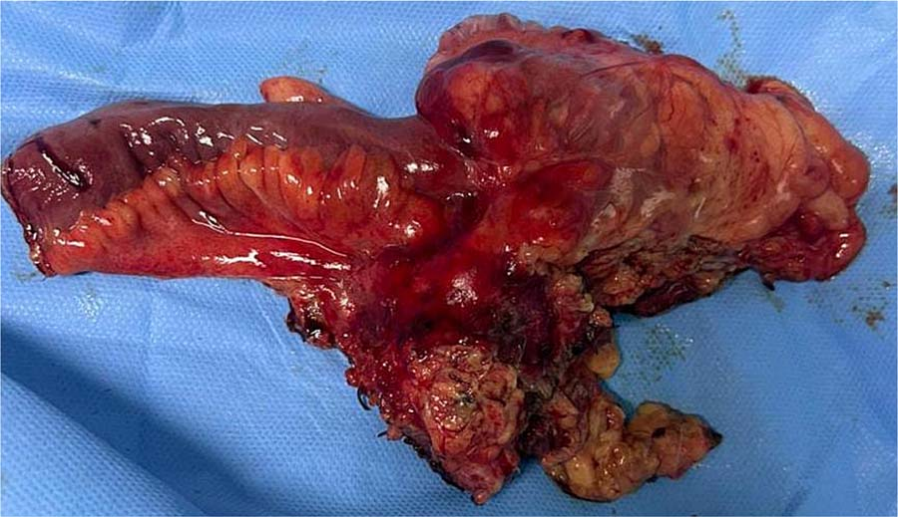
Surgical room.

A 29-year-old patient with no significant medical history presented with right iliac fossa pain associated with diarrhea for 4 years, evolving in a context of preserved general condition. Clinical examination revealed tenderness in the right iliac fossa with a good overall state. The biological assessment was normal. The patient underwent a total colonoscopy, which identified non-specific interstitial colitis. An abdominal computed tomography scan was then performed, suggesting an intraperitoneal mass located in the right iliac fossa, containing calcifications and measuring 37 × 49 mm (Fig. 1). The biopsies were non-contributory. The patient was operated on, and surgical exploration revealed the presence of a small intestine tumor invading the base of the cecum. An ileocecal resection was performed (Fig. 2). Histological examination showed a tumor proliferation of fibroblastic nature with variable cellular density, including areas of hypocellular fibrous collagen. There was also an associated lymphocytic inflammatory infiltrate and calcifications. To rule out the diagnosis of gastrointestinal stromal tumors, the main differential diagnosis, immunostaining with anti-CD117 antibody was conducted. The tumor did not express this antigen. The diagnosis of calcifying fibrous tumor was retained.

## Discussion

The calcifying fibrous tumor is a rare benign mesenchymal tumor [1], typically occurring in young individuals with various locations in the body [2]. It was initially described by Rosenthal *et al.* as a fibrous tumor of childhood with psammomatous bodies. The WHO classification of soft tissue and bone tumors ultimately adopted the term calcifying fibrous tumor. The first reported cases were localized in the subcutaneous tissue and soft parts; subsequently, several other locations have been reported. The localization in the small intestine is rarely described, with only a few cases reported at this site [3]. The calcifying fibrous tumor is classically a tumor of young individuals, with no sex predilection, and age extremes ranging from 1 to 71 years. Clinical presentations are variable. The tumor may be discovered incidentally, present as chronic abdominal pain, or manifest as an acute abdominal syndrome. Imaging is inconclusive; the presence of calcifications within the tumor could be a suggestive element. Differential diagnosis primarily involves stromal tumors. The low cellularity of the proliferation, dense collagenous background, psammomatous bodies, and good delineation of the tumor are arguments in favor of the calcifying fibrous tumor. The immunohistochemical profile of these tumors is characterized by the expression of vimentin and negativity for anti-actin antibodies, S100, cytokeratin, CD31, and CD117. The pathogenesis of this tumor is debated; according to some authors, the calcifying fibrous tumor represents the final evolutionary stage of inflammatory myofibroblastic tumor (IMT). Treatment is surgical. The calcifying fibrous tumor is a rare benign mesenchymal tumor rarely described in the small intestine. It has distinct imaging and histopathological characteristics compared to stromal tumors. In practice, the diagnosis of calcifying fibrous pseudotumor is rarely made before surgical intervention due to unfamiliarity with the entity and often non-contributory preoperative biopsies. Suspicion of stromal tumor often leads to surgical intervention. If the diagnosis of calcifying fibrous pseudotumor could be made through biopsies, due to its benign nature, slow evolution, and potentially morbid nature of surgical intervention, simple surveillance of the lesion through imaging could be considered [4].

## Conclusion

The calcifying fibrous tumor is a benign tumor rarely localized in the small intestine. It can present with several misleading clinical pictures. The diagnosis is histological.
